# Unlocking digital archives: cross-disciplinary perspectives on AI and born-digital data

**DOI:** 10.1007/s00146-021-01367-x

**Published:** 2022-01-12

**Authors:** Lise Jaillant, Annalina Caputo

**Affiliations:** 1 Loughborough University, Loughborough, UK; 2 ADAPT Centre, Dublin City University, Dublin, Ireland

**Keywords:** Born-digital archives, Artificial Intelligence, Privacy, Copyright, Sensitivity Review, Ethics

## Abstract

Co-authored by a Computer Scientist and a Digital Humanist, this article examines the challenges faced by cultural heritage institutions in the digital age, which have led to the closure of the vast majority of born-digital archival collections. It focuses particularly on cultural organizations such as libraries, museums and archives, used by historians, literary scholars and other Humanities scholars. Most born-digital records held by cultural organizations are inaccessible due to privacy, copyright, commercial and technical issues. Even when born-digital data are publicly available (as in the case of web archives), users often need to physically travel to repositories such as the British Library or the Bibliothèque Nationale de France to consult web pages. Provided with enough sample data from which to learn and train their models, AI, and more specifically machine learning algorithms, offer the opportunity to improve and ease the access to digital archives by learning to perform complex human tasks. These vary from providing intelligent support for searching the archives to automate tedious and time-consuming tasks.  In this article, we focus on sensitivity review as a practical solution to unlock digital archives that would allow archival institutions to make non-sensitive information available. This promise to make archives more accessible does not come free of warnings for potential pitfalls and risks: inherent errors, "black box" approaches that make the algorithm inscrutable, and risks related to bias, fake, or partial information. Our central argument is that AI can deliver its promise to make digital archival collections more accessible, but it also creates new challenges - particularly in terms of ethics. In the conclusion, we insist on the importance of fairness, accountability and transparency in the process of making digital archives more accessible.

## Introduction

Co-authored by a Computer Scientist and a Digital Humanist, this article examines the challenges faced by cultural heritage institutions in the digital age, which have led to the closure of the vast majority of born-digital archival collections. It focuses particularly on cultural organizations such as libraries, museums and archives, used by historians, literary scholars and other Humanities scholars. Most born-digital records held by cultural organizations are inaccessible due to privacy, copyright, commercial and technical issues. Even when born-digital data are publicly available (as in the case of web archives), users often need to physically travel to repositories such as the British Library or the Bibliothèque Nationale de France to consult web pages. Provided with enough sample data from which to learn and train their models, AI, and more specifically Machine Learning algorithms, offer the opportunity to improve and ease the access to digital archives by learning to perform complex human tasks. These vary from providing intelligent support for searching the archives to automate tedious and time-consuming tasks.

In particular, we focus on sensitivity review as a practical solution to unlock digital archives. The conclusion insists on the importance of fairness, accountability and transparency in the process of making digital archives more accessible.

How can we make born-digital data more accessible? And what is the role of Artificial Intelligence in unlocking “dark” archives closed to users? The golden age of bulky paper archives is behind us. Emails have replaced letters, PDFs and Word documents have replaced paper reports, and online encyclopaedias have replaced heavy paper volumes. Because “born-digital” archives are natively supported on digital media, they seem at first sight cheaper to maintain and easier to access. However, the relentless growth of internet, which has expanded to include the web of context first, with semantic web and contextualised content, and the web of things lately, encompassing all the content generated by a myriad of small "smart" devices, is resulting in the massive generation of born-digital data.

Archives used to be the prerogative of institutions and companies that had the resources and time to dedicate to them. Traditionally, public archival institutions collected government and administrative records. The National Archives of France, for example, were created in the 1790s to collect the archives of central institutions suppressed by the French Revolution, as well as the archives of ecclesiastical establishments of the diocese of Paris. Ministry archives were added to this list in the nineteenth century. It was not until the post-Second World War that new fields of collection developed: business archives, but also personal and family archives. With the feminist and cultural-culture movements of the 1960s and 1970s in France and other Western countries, personal archives became more diverse, expanding their scope from a narrow focus on white “great men” towards women and minorities (Dumont-Johnson 1975; Quinn 1977; Mason and Zanish-Belcher 2007).

With the popularization of personal computers in the 1980s and mobile devices at the turn of the twenty-first century, individuals started producing a tremendous amount of data. Archives have become more “personal,” either because they capture portions of an individual’s life, or because they hold personal information. Tech companies such as Google and Facebook have become experts at using these personal data to analyze our habits and predict our future behavior. For Zuboff (2019), Google and Facebook are the archetypes of “surveillance capitalists,” at the center of an economic system that “unilaterally claims human experience as free raw material for translation into behavioral data” (2019). According to Zuboff, our data are fueling an economic machine that has little concern for the privacy of individuals.

As non-profit organizations, libraries and archives have very different concerns from tech companies. The vast majority of the born-digital records held by cultural organizations are inaccessible due to privacy, copyright, commercial and technical issues. Even when born-digital data are publicly available (as in the case of web archives), users often need to physically travel to repositories such as the British Library or the Bibliothèque Nationale de France to consult web pages. Users who seek access to their personal data held by Google, Facebook and other companies often face multiple obstacles—including a lack of response following data access requests (Verborgh 2019). It is difficult to know what firms collect about their users, and what they do with these data. Locked behind walls, born-digital data have become “dark” for the vast majority of users.

In this digital world, it is increasingly important to re-think and redesign the way we access information. Artificial intelligence offers the promise to make born-digital archives more accessible—for example by identifying sensitive information, which would allow archival institutions to make non-sensitive information available; or by flagging documents as relevant to a particular search query. Artificial intelligence (AI) is a large concept designating the creation of intelligent machines that can simulate human thinking capability and behaviour. AI encompasses a variety of approaches, but it is with machine learning, a sub-branch of AI concerned with learning from data without being programmed directly, that AI has become a mainstream technology with the promise of revolutionising multiple sectors. So much so, that the two terms are often used interchangeably. In the context of digital archives, this technology can lead to learning from existing corpora and annotated datasets to automatise and simplify daunting tasks, like the manual review of sensitive or copyright material, or providing support to users in searching and making sense of these archives. However, the opaque mechanisms through which these learning algorithms train their models must be subject to scrutiny, otherwise, pitfalls in the data from where they learn can be easily translated into wrong decisions and skewed representation of the reality.

This promise to make archives more accessible does not come free of warnings for potential pitfalls and risks. First, there are inherent errors that lurk behind many algorithms. For example, ePADD (an open-source software to manage email archives) is not always effective at detecting sensitive terms. Working in the archival emails of the poet Wendy Cope, Callum McKean (Lead Curator, Contemporary Archives and Manuscripts, British Library) noticed that ePADD flagged “mushroom” in Cope’s shopping list.^1^ The lexicon classes “mushroom” as a drug, leading to false positives. Second, entrusting artificial intelligence to make decisions in complex situations can lead to ethical and social problems.^2^ Due to the “black box” nature of AI, it is difficult to understand why the machine makes the decisions it makes. Moreover, the data used to train AI systems may contain biases—for example, since White men are over-represented at the most senior levels of government and other sectors, documents by women or ethnic minorities could be flagged as less important. Risks of biased, fake, or partial information are intertwined with AI.

In this article co-authored by a Computer Scientist and a Digital Humanist, our central argument is that AI can deliver its promise to make digital archival collections more accessible, but it also creates new challenges—particularly in terms of ethics. “Explainable AI” (which allows human users to comprehend the results created by machine learning algorithms) is becoming essential to understand how the machine came to particular decisions. To address these new challenges, collaborations between archivists, Digital Humanists and Computer scientists are essential. Indeed, the major challenges of our time—from global warming to social inequalities—cannot be solved within single disciplines. The same applies to the challenge of inaccessible data. The UK/Irish network AURA (Archives in the UK/ Republic of Ireland and AI) and the UK/US network AEOLIAN (AI for Cultural Organizations) bring together Humanities scholars, Computer scientists, archivists, librarians and museum professionals.^3^ Other initiatives—such as AI for LAMs—adopt a more specialized approach targeted to staff in Libraries, Archives and Museums.^4^ These activities show that there is a tremendous appetite for exchanges on the topic of AI applied to digital archives, but also for specific case studies. Whereas AI has become mainstream in a wide range of sectors, it is still at the experimental stage in libraries and archival institutions, and there is “a lack of compelling case studies” (Rolan et al. 2019).

This article starts with an overview of the challenges faced by archival institutions in the digital age, which have led to the closure of the vast majority of born-digital collections. We focus particularly on cultural organizations such as libraries, museums and archives, used by historians, literary scholars and other Humanities scholars. What would access to born-digital materials in these archives look like? And what would it look like to use Artificial Intelligence in archives? In particular, we focus on sensitivity review as a practical solution to unlock digital archives. In the conclusion, we insist on the importance of fairness, accountability and transparency in the process of making digital archives more accessible.

## Privacy and data protection

“Dark” archives refer to collections that are closed to users for a wide range of reasons. Privacy concerns and the need to comply with data protection laws often lead to the closure of entire born-digital collections, or to highly restrictive measures that drastically decrease the number of potential users (Jaillant 2019; Baron and Payne 2017). In Europe, the 2018 General Data Protection Regulation gives to data subjects the right to obtain “the erasure of personal data concerning him or her without undue delay”—a right limited by “archiving purposes in the public interest” (Art. 17 GDPR). In practice, many archival institutions prefer to close entire collections rather than appeal to the public interest. Likewise, the 2018 Data Protection Act, which applies the GDPR’s standards in the UK and replaces previous data protection legislation, has often been interpreted in a very restrictive manner. Getting access to born-digital data in British archives is often impossible—especially for collections centered on living people.

For example, literary scholars and other users interested in the archive of the British poet Wendy Cope (born in 1945) are still unable to access any information on the British Library (BL) catalogue “Explore Archives and Manuscripts.” Yet, the BL acquired the Cope archive a decade ago, in 2011. The hybrid archive, encompassing material in both paper and electronic form, comprises 15 large storage boxes as well as Word files. The collection also included a large number of emails, initially estimated at around 40,000 (Flood 2011; Some Sort of Record 2011). As Rachel Foss (Head of Contemporary Archives and Manuscripts at the British Library) points out:

It was quite a complex process to capture the emails given how they were created and held by the depositor, in multiple folders, some duplicates and invisible files etc. This was several years before we began to work with [the open access software] ePADD and without being able to use a processing tool such as this, it’s a bit of guess. As it turned out, there were some errors in double-counting across folders. The number of emails in the collection is 25,556.^5^

At the request of the British Library, Wendy Cope was encouraged to weed out her email collection to help with appraisal (the selection of records for permanent preservation). In a 2019 presentation, Callum McKean said that the British Library had asked Cope to “sort through the content she’d like to pass to us and dispose of everything she didn’t want to pass to us.” He added: “Cope actually found the process quite tedious and arduous, and as expected there would still be significant Data Protection challenges with such a vast and personal archive.”^6^

The BL has done extensive work to preserve this archive and to try to make it more accessible, with limited success so far. In  Pledge and Dickens (2018) article, Jonathan Pledge and Eleanor Dickens (who work as curators in the contemporary archives department) explained the BL’s plans to make selected born-digital records available in reading rooms. Callum McKean also pointed out that the overall plan of the British Library was to give access to emails, but on a highly restricted basis. Researchers would be “granted access to text files only in PDF/A, one file at a time, in a way analogous to the way paper material is made available.”^7^ To this day, however, the Wendy Cope archive is inaccessible to users.

The lack of discoverability of the Wendy Cope collection (with no Finding Aid publicly available on the catalogue) means that very few users will know that the British Library holds this archive. No information is provided about the estimated date when the archive will be made available. Will the collection cease to be “dark” in the near future? And if so, what will be the conditions of access? Users do not currently have the option to download and analyze data from the archive. The policy to provide deliverable units (one digital file at a time) is reminiscent of Terry Cook’s warning that “paper minds” cannot deal with “electronic realities” (Cook 1994). In other words, the processes used to give access to paper archives are also applied to born-digital files. This has an impact on research methods: for example, close reading can be used to analyze individual archival emails, but quantitative methodologies could be applied if researchers had access to larger amounts of data.

Requiring users to travel to reading rooms is still another constraint on access, as the closure of cultural organisations during the COVID-19 pandemic has shown. Even when libraries and archives are open, requiring users to consult documents onsite excludes people who cannot travel for health reasons, family responsibilities and lack of funding. Again, the process adopted here is that of the paper archive: only a small portion of paper collections have been digitized due to many reasons (including lack of funding, fragility of documents, and the size of collections) and it is common practice to ask users to travel onsite. In the rare cases when emails and born-digital records are available, users also need to travel onsite. For example, Yale University’s Beinecke Library gives onsite access to selected electronic files from the archival collection of the British writer Peter Ackroyd. Yet, these documents could be shared online—for example as part of a secure online system accessible to registered patrons.

Archival collections often close entire collections due to data protection concerns. To give just an example, the email archive of the British novelist Ian McEwan at the Harry Ransom Center in Austin, Texas is not currently available to researchers. But are these institutions mostly concerned about the rights of data subjects to privacy? Or are they worried about their own reputation and the potential risk of a lawsuit? Comparing their practices with that of tech giants such as Google is illuminating. Google has been repeatedly fined for data protection breaches, including a record €100 m fine in 2020 for GDPR violation in France.^8^ So far, these repeated fines have had little impact on Google’s determination to gather as much data as possible about users, and to monetize these data. While the GDPR supports “data minimization” and the principle that personal data shall be “adequate, relevant and limited to what is necessary in relation to the purposes for which they are processed” (Art. 5), Google’s business model relies on data maximization purporting to serve the public interest to be informed.

Archival repositories too rely on data maximization since their core function is to collect, organize, preserve, and make accessible cultural heritage materials to both the academic research community as well as the public. But they adopt a much more risk-adverse position than Google.^9^ In the balance between data subjects rights and public interest, they privilege rights to privacy (and their own interest in avoiding reputational and legal challenges). In contrast, Google privileges the public interest and freedom of information (and their own commercial interests). We argue that these two radically opposed attitudes are equally wrong-headed. Google—a company that has largely replaced archives and libraries’ traditional function to provide search and access to information—has too often ignored individuals’ legitimate right to privacy. And archival institutions have neglected users’ legitimate right to access information. Archives and libraries have a role to play in challenging powerful tech giants, but they cannot play this role if they do not fully embrace open data respectful of privacy. Making data more open does not mean that everything has to be accessible—it is legitimate to close down parts of collections that are particularly sensitive. As discussed later in this paper, AI can be a useful support to the cumbersome task of manually identifying sensitive content, yet it is still prone to error. Broader is the definition of sensitive information, which includes legal agreements, classified or privileged communications, and confidential or ethical information (Woods and Lee 2015). Hence, the requirement to open datasets and make them searchable contrasts with the necessity to preserve individual rights to privacy and, more generally, for reasons connected to national security and international relations. But closing entire collections for an indeterminate period of time is not ethical, since archives in publicly funded organizations are meant to be open to the public. It also marginalizes archival institutions at a historical moment when tech companies exercise huge control over our digital memory by holding vast amounts of personal data (think of all the photo files that Facebook has gathered, for example).^10^

## Copyright

In addition to data protection laws, cultural organisations need to comply with copyright legislation which also has an impact on accessibility. In the UK, publishers are required to deposit electronic copies of their publications in legal deposit libraries such as the British Library, but access to these works is severely restricted by the Legal Deposit Libraries (Non-Print Works) Regulations 2013. Access is onsite, and only one reader can access the same electronic publication at the same time. Copyright also explains why web archives often need to be consulted onsite, since libraries and archives are not always able to trace copyright holders to obtain permissions to share the materials more widely.

To access French web archives, users need to obtain a reader’s pass and use the reading rooms at the Bibliothèque Nationale de France (BNF) to access web pages that were once publicly available. Getting a reader’s pass can be an intimidating process, especially for those who do not belong to specific categories (academics, postgraduate students, journalists and LAM professionals). A personal interview with a BNF staff member is required, to make sure that the applicant is doing research and has a legitimate reason to come to the BNF. This restricts access to a minority of users: those who can travel to Paris, can express themselves confidently in French, and can provide required justifications. To make web archives more accessible, the BNF allows distant access to its own collections from a few legal deposit libraries outside Paris. But provincial users still need to travel onsite to look at these documents.

Researchers are often unable to use computational methods to analyze born-digital records locked within libraries and archives. As Jane Winters puts it, there is a “background of increased expectation” (Winters 2017) from users who expect open access to data. This is true of ordinary users, but also of more specialized researchers who want to use materials for text and data mining (TDM). In a 2020 presentation entitled “No Text, no text mining,” Beatrice Alex gave examples of difficulties that scholars face to get access to data.^11^ This includes (1) data which are part of a large collection, and it would be too laborious to get permission from individual copyright holders, and (2) data which are in copyright and the owner is not willing to share. It should be noted that getting authorizations from copyright holders is not always necessary. Since 2014, the UK has provided a copyright exception for TDM for non-commercial research, enabling research on copyrighted material (Intellectual Property Office 2014). If a researcher has the right to read a copyrighted document under the terms of the licensing agreement with the content provider, they also have the right to copy the work for the purpose of non-commercial TDM. However, the law still makes it impossible to do TDM on non-print materials, for example born-digital records in legal deposit libraries such as the British Library or the National Library of Scotland (Gooding et al. 2019). As Sarah Ames and Stuart Lewis put it, this restrictive legislation leaves “challenges for libraries as they seek to make collections available at scale” (Ames and Lewis 2020).

Cultural institutions routinely reject requests to use computational methods on copyrighted twentieth-century materials because the owner is not willing to share or cannot be contacted.^12^ As Melissa Terras argues, “copyright has created a digital dark age where the most powerful tools for cultural analysis are blind between 1910 and the rise of social media.” Terras adds that libraries and archives are often too risk-adverse:

I think the blanket “no” should be replaced by risk assessment, because this should be about institutions understanding their capacity for risk. They should ask “what’s the worst thing that can happen?” I’ve read that only seven libraries have been taken to court in the UK. And if they’re more worried about reputational risk than the benefit they might bring to their audience, there is a problem. Because is it really copyright—the right of the author—that you’re worried about? (Mackinlay 2021)

In short, copyright can be used as an excuse to close entire collections—instead of doing a risk assessment to evaluate the risks of legal issues with copyright holders (which are typically very low).

Since access to twentieth-century materials is so complicated, it often makes sense for Digital Humanists to focus on data in the public domain, especially nineteenth-century collections that were mass-printed and have a standardized form. For example, living with machines (a major project funded by the UK Arts and Humanities Research Council) uses nineteenth-century materials including newspapers, census records, maps and other sources, to understand the impact of the Industrial Revolution. The multi-disciplinary project team is developing new methods in data science^13^ to allow researchers to study these collections on a massive scale (Living with Machines 2020).

Among other sources, living with machines relies on digitized and online map collections provided by the National Library of Scotland (NLS), an institution that has done pioneering work in publishing data openly and in reusable format. The NLS’s new digital scholarship service focuses on providing digitized collections as datasets, metadata collections, audiovisual material, map and spatial data, and organisation data. “Digitised print collections are only part of a much bigger landscape within which digital scholarship operates” (Ames and Lewis 2020). These data are openly accessible via an online platform, the Data Foundry.^14^

## What would access to born-digital materials in archives look like?

The National Library of Scotland offers a good model for providing access to digitized materials as datasets, as well as born-digital materials—a model that could be replicated in other institutions. Let us take the example of the Edinburgh Ladies’ Debating Society dataset, which is part of the digitized collections. The collection includes a total of sixteen volumes of two Edinburgh journals, *The Attempt* (1865–74) and its successor *The Ladies’ Edinburgh Magazine* (1875–80). These publications were produced by a leading Edinburgh women’s club, which existed from 1865 to 1935. At a time when women were often confined to the private sphere, the Debating Society offered an opportunity to share opinions on education, suffrage, health and welfare. The magazines contain articles about women’s access to higher education and salaried work, women’s suffrage and rights, religion, as well as literary criticism, fiction and poetry. The NLS received copies of the journals in 1936, shortly after the Debating Society was dissolved. The manuscript minutes of the society were also gifted to the Library.^15^

The dataset includes a total of 6354 xml files at page level and 6354 image files, with METS metadata files at item level.^16^ These metadata files feature contextual information including the date when the item was digitized, the technical material used to capture the images, and the method used for transcriptions—which were generated from Optical Character Recognition (OCR) performed by the National Library of Scotland. In total, the collection includes 259,829 lines and 2,654,641 words. Users have the option to download everything or just a sample of the dataset for initial evaluation.

Most of the other resources available on the Data Foundry website share the same characteristics as the Edinburgh Ladies’ Debating Society dataset. They were produced as part of the mass digitization programme underway at the NLS. Indeed, the Library’s strategic aim is to have one third of its total collections available in digital form by 2025 (National Library of Scotland 2015). Moreover, these resources are a response to the “collections as data” movement which has encouraged cultural heritage organizations to present collections in machine-readable formats. These collections originated in paper, and do not present any issues with privacy/ data protection and copyright. Not surprisingly, materials from the nineteenth century and earlier are over-represented, while born-digital records are under-represented. At the time of writing (April 2021), there are no email collections or web archives on the Data Foundry website. Among the few born-digital records available are organisational data such as transactions over £25,000 and government procurement information, available in CSV files.

The quasi-invisibility of born-digital records on the Data Foundry platform contrasts sharply with the exponential rise in these records at the National Library of Scotland. In 2020, the NLS reported that over 5.2 million e-journals and e-books had been deposited via non-print legal deposit into shared infrastructure (Ames and Lewis 2020). As in the case of web archives, copyright is a major obstacle on the road to publish data openly. With “dark” archives closed to researchers on the one side, and openly accessible platforms on the other side, the current situation is extremely polarized.

Perhaps it is time to imagine more nuanced models that would provide access to selected users who fulfil certain conditions. Special Collections libraries routinely ask researchers to provide their ID before getting access to (print) archival records in reading rooms. Other documents are sometimes required—including letters of introduction or recommendation (for example to access very valuable manuscripts at the British Library),^17^ and signed agreements to comply with the library’s internal rules and with the overall legal framework. Before researchers use their digital cameras to copy materials, they often need to sign a form to confirm that they are familiar with copyright and data protection laws. These measures are in place to protect the collections and to avoid legal concerns. For instance, if a user decides to post copies of confidential or sensitive archival materials on social media, the library would be able to show that the responsibility lies with the user not with them. Libraries need to show that they take their responsibility as custodians of manuscripts and archives very seriously.

Why not adopt a similar access system for born-digital records, especially records that are particularly sensitive, instead of closing entire collections? Users would provide their ID and relevant credentials, as well as sign necessary forms, before getting access to a secure online system to consult email records and other born-digital documents. Ideally, libraries would loan data to researchers for a few days or weeks, with the function to access masses of data instead of having to consult individual items. As in the case of ebooks that libraries loan via protected systems such as Acrobat Digital Editions, born-digital records could be consulted within a secure online system. Researchers would bring their tools to the system, instead of downloading data on their own computers. This approach is currently not taken due to the perceived cost of technical infrastructure to build a secure access system. It would also require a change of processes which, as we have seen, are still focused on paper rather than digital.

Providing access behind a secure online system is a middle-of-the-road model that will not satisfy everyone. On the side of the open access movement, some will argue that less problematic records such as web archives should be made freely available, not locked behind walls. The National Library of Ireland already makes selected archival websites freely available. To protect itself against possible claims from copyright holders and data subjects, the NLI states: “It is the responsibility of the site owner to comply with the Data Protection and Copyright Legislation. The National Library archives these materials in the public interest and we make them available for the purposes of research and private study.”^18^ On the side of a more restrictive approach to access, there will be concerns that no online system will be robust enough to make sensitive born-digital records available. Even if users cannot download data on their own systems, they would still be able to take screenshots of records. Some of these pictures could then be circulated on the web and social media. The cost of building a secure online system will also be an issue, especially for smaller institutions that already struggle to preserve their born-digital collections.

One way of answering these concerns is to point out that providing limited access is better than providing no access at all (Jaillant 2020). The open access movement has played an invaluable role in pushing for more availability and transparency, but it has also scared many cultural organizations that fear they lack appropriate resources and risk-management models to open up their collections. Positions on both sides of the open-versus-closed border are so entrenched that dark archives are arguably becoming darker and darker. Even practices that were widely accepted for print collections (such as providing an approximate date when a collection will be made publicly available) are not the norm for born-digital collections. It is time to design a new approach.

For institutions which cannot afford to build an online system to provide access to born-digital records, other solutions could be envisaged, such as participation in a consortium bringing together libraries and archives (on the model of the HathiTrust, a partnership of academic and research institutions that offers a collection of millions of titles digitized from libraries around the world).^19^ Building a born-digital consortium would be a welcome step towards more accessible collections. It would also show that cultural organizations can get together to build a platform for the public good, instead of letting private companies such as Google dominate the digital environment. Back in 2004, Google Books started digitizing lots of books and, in the case of works already in the public domain, imposed its own download policies and other contractual limitations. Four years later, HaithiTrust was created as a not-for-profit consortium designed to make digitized content accessible to the widest number of users. HaithiTrust focuses mostly on digitized content, and on partner institutions in the United States. This focus on digital materials that originated in published paper form explains why HaithiTrust is more concerned about copyright than privacy and data protection (a central concern in the case of unpublished materials). Its activities are conducted in light of US Copyright Law and with the guidance of the University of Michigan’s Office of the General Counsel.^20^ There is space for a not-for-profit consortium focusing on born-digital content, and on a global community of cultural institutions.

How would such a born-digital consortium work in practice? As in the case of HaithiTrust, members would share the costs of operating services and programs.^21^ Institutions would have the option to share selected born-digital collections via aggregators, i.e. organizations that gather data and make them available within the main consortium website. This publishing model is inspired by Europeana, a leading EU-funded website that offers access to millions of digitized cultural heritage items from around 4000 institutions across Europe.^22^ Users would either be able to log in to the central consortium system as guests or as members of partner institutions. Guests would have the opportunity to:

_search across the entire collection;

_read, view and download content that is “full view”;

_search within content that is “limited access”;

_access content that does not have restrictions, such as works available on the Internet Archive/Wayback Machine which contains 475 billion web pages and other born-digital content.^23^

Those who log in via a partner institution would have access to a wider range of options including creating, saving and sharing collections on specific themes; and getting access to “limited access” content that cannot be made openly accessible on the web (such as email collections). A pilot project would need to be developed first, to explore the best ways to make content available. For archival emails, it might make sense to give access to text files only in a format such as PDF/A. This format is not ideal since it does not preserve all the characteristics of emails (for example the fact that emails are often threaded rather than independent texts). Yet, even a small step in the direction of greater access to born-digital archives would be a great improvement on the current situation characterized by lack of discoverability and accessibility.

## What would it look like to use Artificial Intelligence in archives?

Artificial Intelligence can also be used to find relevant content. Law firms engaged in litigation now routinely use “eDiscovery” tools, relying on AI to make search more effective than traditional keyword search. In addition to finding supporting evidence which is used to prove or disprove a case, eDiscovery can also reveal if evidence has been destroyed or is missing. The global eDiscovery market size is expected to grow from USD 9.3 billion in 2020 to USD 12.9 billion by 2025.^24^ These tools are based on predictive coding, a form of ML that learns from a subset of documents selected by lawyers and attorneys, and then applies what it has learned to a much larger set of documents. Indeed, the algorithms developed on the selection of the documents can then be applied to a huge dataset, making the review process quicker, cheaper, and less complex. Using eDiscovery software does not require advanced technical skills, which explains the popularity of these tools.

Similar tools could be used by scholars to identify relevant content, but relying on commercial off-the-shelf software can be problematic. In his report on Machine Learning and Libraries, Ryan Cordell notes that he has “avoided much discussion of vendor-supplied ML tools, primary because [he does] not, in general, believe they meet the standards of openness, explainability, and adaptability that best practices encourage.” Likewise, Abigail Potter at the Library of Congress is quoted as saying that “there is a mismatch in what is being offered, i.e., full solutions or black box tools, and the needs in cultural heritage for transparency, assessment, auditing and perhaps reprocessing of data” (Cordell 2020). Like archivists who need to participate in the process of assisted review of archival documents, scholars need to engage with ML to understand how and why the machine has selected certain documents within a large dataset.

Engaging with machine learning does not necessarily require advanced training in computer science. Let us go back to the example of the Data Foundry, a website that is accessible to all users—including those without previous programming experience. It offers not only datasets, but also various tools to analyze collections. In the case of the Edinburgh Ladies’ Debating Society dataset, Lucy Havens at the National Library of Scotland created a practical guide to explore the collection using text and data mining, as well as Natural Language Processing (NLP) with the programming language Python.^25^ NLP is a branch of AI concerned with giving machines the ability to understand text and spoken words in much the same way humans can. Examples of NLP tasks include speech recognition, sentiment analysis and named entity recognition which identifies words and phrases as useful entities (such as locations or first names). Havens used named entity recognition to automatically identify male and female names. She then visualized her dataset, to show the number of occurrences of the name “Mary” over time. The step-by-step approach makes it possible for users with even limited technical knowledge to understand how to apply NLP to library collections. Havens also gives advice for further training using the Library Carpentry website and a NLP book openly accessible with a Creative Commons license (Alex and Llewellyn 2020; Bird et al. 2019). While the Edinburgh Ladies’ Debating Society dataset used in this example is out-of-copyright and does not present any issue with data protection and privacy, other collections can be much more complicated to analyze. For example, the language used in emails often relies on shared understanding between the correspondents. This kind of gossipy, informal language makes it difficult for the machine to identify relevant words or phrases—making it necessary to have a human fact checker at the end of the process.

## Artificial Intelligence and sensitivity review

As we have seen, the issue of sensitive and confidential materials is one of the key reasons why so many born-digital collections are unfindable and inaccessible. Artificial Intelligence and Machine Learning can be used to review huge numbers of digital files, and identify problematic materials. In a 2020 presentation, Steve Rigden, Digital Archivist at the National Library of Scotland, talked about the role of AI in identifying sensitive materials in digital collections at the NLS. He insisted on the role of archivists in reviewing data and making final decisions. Indeed, archivists need to identify datasets; identify the most effective algorithms to apply; test and refine the models for testing data, to give the machine what it needs to learn; and further refine and retest. Archivists do not have to be proficient in technical aspects of AI, Rigden argued, but they do have to have the interest to engage with the development of such tools as advocates, as advisers and as testers. In other words, they need to actively participate in this process of “assisted review” of archival documents.^26^

The capability to process and automatically classify big data presents one of the biggest opportunities for the use of AI for born-digital archives. The growing awareness and legal implication resulting from the implementation of different Data Protection Acts (from the European GDPR to the UK Data Protection Act 2018) clashes with the more than 100 worldwide different implementations of the freedom of information laws and acts, which instead are designed to ensure access to government documents. Hence, to unlock born-digital archives means first of all, being able to correctly identify and classify sensitive and personally identifying information (PII).

PII refers to any information that can uniquely identify an individual, from names, phone and security numbers, to birth and medical data. PII involves classified and legal information as well as data resulting from research experiments. The definition of PII can be elusive, to include even information that cannot be directly ascribed to an individual, such as search logs and IP addresses, since it can still lead to the identification of individuals if appropriately mined. For example, Sweeney (2002) demonstrated how it is possible to re-identify individuals by cross-linking datasets, even when previously anonymized, simply looking at common attributes such as ZIP codes, birth of date and gender.

Typical information retrieval systems, such as web search engines, are processors of information: in an attempt to optimize both precision and recall, they follow the policy that if something is available then it is also findable (Olteanu et al. 2021). The right balance between openness and protection can be reached by revising this approach to data processing and consumption. Protect-and-search and search-and-protect are two possible paradigms to solve this problem. However, as Olteanu et al. (2021) point out, we want to preserve sensitive information not only from human sight, but also from the search engine. Hence, a new perspective on the problem can favor new architectures that embed both relevance and sensitivity.

Irrespective of the paradigm of choice, we consider two areas of intervention when facing sensitivity in accessing born-digital archives: the identification and quantification of sensitive information.

### Identify

First, the need to identify what is sensitive. This task can be broad, as general text classification, or very specific, to surgically pinpoint portions of texts that could disclose sensitive or personal information. Text classification is a classical and explored area of machine learning (Sebastiani 2002) where a set of characterizing features extracted from the documents are used to predict the classes, or categories, of a document. This is a typical problem of supervised learning, where a model is *trained* by looking at a pre-annotated collection of documents tagged with the correct classes. Training a model usually means learning a mapping function between the input (features representing the documents) and the output (classes). The function can be a simple linear combination of feature weights or a complex model, as the ones captured by deep neural networks, with multiple nested layers of activation functions and thousands of parameters. One crucial aspect of this class of algorithms is defining the set of features that adequately capture the correlation between input and output. In text classification, features are usually extracted directly from the text in form of keywords, i.e. the single terms constituting the text.

However, as McDonald et al. (2020) note, sensitivity review is not a *topic-oriented task* where keywords are useful to identify the topic of a document, but rather a *who said what about whom* task, where is the relationships between terms and entities in the discourse, in addition to the single keywords, to disclose sensitive information.

Therefore, this set of very basic features is often enriched with more sophisticated ones coming from Natural Language Processing and Information Extraction pipelines. Syntactic information, (such as Part-of-Speech (POS) tags), structural information (such as headings of sections and tables) and whole sequences of words (also referred to as n-grams) are often employed to capture *composite* sensitivity, which can result from the combination of multiple types of sensitivity. Moreover, to overcome the ambiguity of language, where polysemy and synonymy result in topic drifting and mismatch, and at the same time capture contextual semantic information, word embedding features coming from distributional semantic models can replace or be juxtaposed with simple keywords. McDonald et al. (2020) combined all of these (terms, POS n-grams, and word embeddings) into a SVM classifier for categorizing a collection of 3,801 government documents as either sensitive or not-sensitive. The authors showed that the inclusion of the semantic features (word embeddings) increases the accuracy of the classifier by 9.99% with respect to baseline approaches.

Classification is not always binary (sensitive vs. non-sensitive) and sometimes a graded notion of sensitivity adheres better to the underlying task. In an empirical research aimed to provide a “more robust theory of official secrecy that can both account for variation in classification practices and inform more effective regulation”, Souza et al. (2016) worked with approximately one million diplomatic cables from the 1970s classified as “secret,” “confidential,” “limited official use,” or “unclassified.” The authors noticed that “top secret” documents, although limited in quantity, were not included in the collection. Document text was processed with standard tokenization and normalization techniques. However, additional information reflecting the structure of the documents and the originating fields of the features (sender/recipient, subject, body, etc.) were embedded in the representation. The authors experimented with some standard classification algorithms built on weighted feature vectors. Observations from the outcomes led to the conclusion that dates were of limited use for this classification task, while words in the *body* field were the most valuable to discriminate sensitive information. Overall, the best performance was achieved when all the features vectors worked in combination. Working with these graded sensitivity classes led also to another finding. Considering secret and confidential documents, the classifier achieved notable improvements. However, the “limited official use category” led to less clear-cut results, reflecting the nature of these documents, so broad even to elude a formal definition.

Another approach to sensitivity is through the redaction of sensitive/personal information (Woods and Lee 2015). For this purpose, digital forensics tools can be utilised to select candidates for automatic reduction. Part of an open-source effort to the automatic redaction of sensitive documents, BitCurator offers a bulk extractor functionality that lexically analyzes text looking for sensitive features, such as email addresses, phone numbers, and other PII.

Although extensive research has been conducted in the area of privacy-preserving data publishing, much of this work was directed towards relational and statistical data, textual data remains a relatively unexplored area (Fung et al. 2010). Focusing specifically on this type of data, Sánchez and Batet (2016) propose an algorithm for document sanitization that mimics human judgment in assessing the document sensitivity. The assessment quantifies the risk that a set of terms poses to disclose sensitive information by means of inference over a knowledge base that captures sensitive information. As the authors point out, the identification of the appropriate knowledge base is crucial to strike the right balance between domain specificity and model generalisation.

### Quantify

Notwithstanding the impressive results in accuracy, AI algorithms for sensitivity review, either in the form of classification or redaction, are not exempt from failure. In many classification tasks, this may not represent a problem. However, due to legal requirements and the potential severe consequences arising from inadvertently leaking sensitive and private information, a greater care must be taken in sensitivity classification.

Information retrieval has a long history of evaluation that led to the definition of paradigms and metrics aiming at quantifying the capabilities of these systems in retrieving *relevant* information. Metrics of retrieval goodness are usually expressed in terms of precision and recall. However, sensitivity requires other types of considerations, outside the mere topical relevance of documents, that balance the need to access information with the risks involved by disclosing information sensitive in nature, up to the evaluation of impact of worst case scenarios (Olteanu et al. 2021).

Reaching perfect accuracy is impossible. There is an underlying misconception that human annotations provide a gold standard and an upper bound to what AI algorithms can achieve. However, this idea is challenged by the actual quantity of information that annotators can actually review, combined with presentation and cognitive bias that can result in suboptimal sensitivity classification. A symbiotic cooperation human/machine could be the preferred approach, where human scrutiny is not replaced by their algorithmic counterpart, but boosted. McDonald et al. (2020b) investigate the impact on human sensitivity reviews when assisted by automatic classification algorithms. They analyzed the effect of accuracy and prediction confidence levels on (1) the number of documents that human judge and (2) the time they spend to formulate the judgment. The authors’ findings highlighted the value of digital assisted sensitivity review on both speed and quantity. First, sensitive documents required a longer time for being analyzed compared to non-sensitive documents. Second, the reviewer accuracy and speed increased considerably when provided with automatic prediction (+ 37.9 and + 72.2%, respectively). Third, the classification confidence levels impacted the reviewer–classifier agreement: when both agreed, this results in quicker decision while disagreement results in longer overhead for the reviewer.

An alternative approach is to incorporate the humans’ reviews inside the training cycle, hence thinking about ML solutions not as one-shot products, but as living digital assistants that learn side-by-side human reviewers. This adaptability suits particularly well those situations where the sensitivity types are not known a priori (McDonald et al. 2020a). An implementation of this Technology Assisted Review (TAR) is achieved by adopting active learning strategies. The starting point is a seed set obtained by manually annotating a pool of documents satisfying a given query. This seed is used to initially train the algorithm. After this initial stage, the system engages in an iterative process where new predictions are generated for a new set of unlabeled documents, which are then submitted to the human reviewers who generate new manual labels. The original training set is hence expanded with these new training examples, and a new cycle of training begins. This approach has the competitive advantage of reducing the number of labelled documents required to achieve the same classification performances (McDonald et al. 2020a), characteristics particularly appealing when these technologies are to be deployed on new collections.

Overall, these results suggest that the technology is here to stay. Even if “there is no completely automated solution” and “human input is still required at all stages”, The National Archives (2016) highlights the importance of technology-assisted reviews as a way “to understand, value and prioritize born-digital records, as well as reducing the volume needing to be manually reviewed”.

## Conclusion

This paper has argued that AI can deliver its promise to make digital archives more accessible, but it also creates ethical challenges. While the latest achievement obtained by AI in natural language processing, computer vision, machine translation, and the likes, would have not been possible without the ingestion of huge datasets from which training and learning these models, there are undeniable risks generated by this blind supply of data. The most notable example is the bias in the representation of concepts, from which relations like “man is a computer programmer as woman is a homemaker” can reinforce stereotypical views represented in the data (Bolukbasi et al. 2016). In addition, misrepresentation of minority and social views can lead to problematic inference and eventually decision-making processes. Jo and Gebru (2020) take inspiration precisely from archives and libraries, as domains with a well-established language and procedures for collecting data that challenges historical and representation bias. The risk of adopting a blind approach to AI can defeat this purpose. Instead, a framework of AI governance informed by well-developed language and procedures of consent, power, inclusivity, transparency, and ethics and privacy, inspired by consolidated practices among archivists as well in social science, historians and anthropologists, should drive the adoption of AI. Unlocking digital archives requires cross-disciplinary collaborations, but also close attention to ethical principles.

## Footnotes

1 Interview with Callum McKean, 28 May 2021, as part of the AURA project (www.aura-network.net).

2 The scholarship on AI and ethics is extensive. It is useful to start with the definition of ethics as encompassing five principles (transparency, justice and fairness, non-maleficence, responsibility and privacy) (Jobin et al. 2019). See also Hagendorff 2020; Coeckelbergh 2020; Véliz 2020.

3 See www.aura-network.net and www.aeolian-network.net, accessed 16 April 2021.

4 See https://sites.google.com/view/ai4lam, accessed 16 April 2021.

5 Email to author, 22 April 2021. For more information about ePADD, see https://library.stanford.edu/projects/epadd, accessed 26 April 2021; and Schneider et al. 2019.

6 C McKean, Processing E-mail at the British Library, presentation, workshop Born-Digital Archive and Digital Forensic—Where are We Now?, 15 March 2019, School of Advanced Study, University of London.

7 Id.

8 Délibération SAN-2020–012 (2020), https://www.legifrance.gouv.fr/cnil/id/CNILTEXT000042635706, accessed 16 April 2021.

9 Ryan Cordell has argued that this cautious and slow approach in relation to data allows libraries to avoid “destructive ideologies of technological implementation,” focusing on “building rather than breaking” (Cordell 2020).

10 See Ovenden 2020.

11 B Alex, No Text, No Text Mining, AURA workshop 1, November 2020, online, https://www.aura-network.net/2020/12/21/workshop-1-bea-alex/, accessed 16 April 2021.

12 Id.

13 Data science extracts useful insights from data. It is an interdisciplinary field that merges computer science, statistics and other disciplines.

14 https://data.nls.uk/, accessed 16 April 2021.

15 https://data.nls.uk/data/digitised-collections/edinburgh-ladies-debating-society/, accessed 19 April 2021.

16 METS stands for “Metadata Encoding and Transmission Standard” (developed by the US Library of Congress).

17 See https://www.bl.uk/help/access-manuscripts-and-archives, accessed 26 April 2021.

18 https://www.nli.ie/en/web_archive.aspx, accessed 26 April 2021.

19 https://www.hathitrust.org/, accessed 26 April 2021.

20 https://www.hathitrust.org/copyright, accessed 23 July 2021.

21 https://www.hathitrust.org/Cost, accessed 23 July 2021.

22 See https://pro.europeana.eu/share-your-data/process and https://pro.europeana.eu/about-us/mission, accessed 23 July 2021.

23 https://archive.org/about/, accessed 3 May 2021.

24 https://www.marketsandmarkets.com/Market-Reports/e-discovery-market-11881863.html, accessed 3 May 2021.

25 https://data.nls.uk/wp-content/uploads/2020/10/Exploring_Ladies_Edinburgh_Debating_Society.html, accessed 3 May 2021.

26 S Rigden, Sensitivity Review and Access to Digital Materials at the National Library of Scotland, AURA workshop 1, November 2020, online, https://www.aura-network.net/2020/12/21/workshop-1-steve-rigden-sensitivity-review-and-access-to-digital-materials-at-the-national-library-of-scotland/. See also R Oliva, Understanding Sensitivity: A First Step Towards Automating Sensitivity Review, ARCHIVES, ACCESS AND AI conference, January 2021, online, https://www.poetrysurvival.com/presentation-slides-archives-access-and-ai-conference/, accessed 3 May 2021.
